# Protocol for efficient solid-phase synthesis of peptides containing 1-hydroxypyridine-2-one (1,2-HOPO)

**DOI:** 10.1016/j.mex.2020.101082

**Published:** 2020-09-28

**Authors:** Danah Al Shaer, Beatriz G. de la Torre, Fernando Albericio

**Affiliations:** a KwaZulu-Natal Research Innovation and Sequencing Platform (KRISP), School of Laboratory Medicine and Medical Sciences, College of Health Sciences, University of KwaZulu-Natal, Durban 4041, South Africa; b Peptide Science Laboratory, School of Chemistry and Physics, University of KwaZulu-Natal, Durban 4001, South Africa; c Institute for Advanced Chemistry of Catalonia (IQAC-CSIC), 08034 Barcelona, Spain; d CIBER-BBN, Networking Centre on Bioengineering, Biomaterials and Nanomedicine and Department of Organic Chemistry, University of Barcelona, 08028 Barcelona, Spain

**Keywords:** Fe (III) chelators, Siderophores, Solid phase peptide synthesis, 1-Hydroxypyridine-2-one, Trifluoromethanesulfonic acid (TFMSA)

## Abstract

• Metal chelation has found many applications that directly affect human's life.
• Natural siderophores are one of the most potent chelators for Fe (III)
• 1-Hydroxypyridine-2-one (1,2-HOPO) (Fig. 1a), which is shown in 4-carboxy-1-hydroxypyridin-2-one (1,2-HOPO-4-COOH) (Fig. 1b), is a moiety that electronically resembles the hydroxamate group found in natural siderophores (Fig. 1c). Of note, 1,2-HOPO moiety is present in the natural siderophore cepabactin [1]
• Synthesis of 1,2-HOPO containing chelators has been carried in solid phase using carboxylic acid derivatives of 1,2-HOPO and required the protection of the reactive hydroxyl group usually with benzyl group (Bzl). After the peptide elongation, the Bzl group has been removed on the same solid phase using a bit harsh conditions: 0.1 M BBr_3_ in DCM for 60 min [2], 10% HBr in AcOH for 14 h [3]; in solution: 1 M BCl_3_ in DCM for 2 d [4], 50% HCl in AcOH for 4 d [5], H2-Pd/C, AcOH-MeOH [6].
• First of all, a method for the incorporation of the 1,2-HOPO-4-COOH through its carboxyl group into the peptide backbone without protecting the N-OH is proposed (the presence of the carboxyl group facilitates the attachment).
• Furthermore, in the cases that Bzl protection is required for the N-OH, a friendlier method for removing the Bzl is described. The removal of the Bzl is done concomitantly to the global deprotection and cleavage of the peptide from the resin using TFA- TFMSA-H_2_O (8:3:1).

## Graphical abstract

**Figure:**
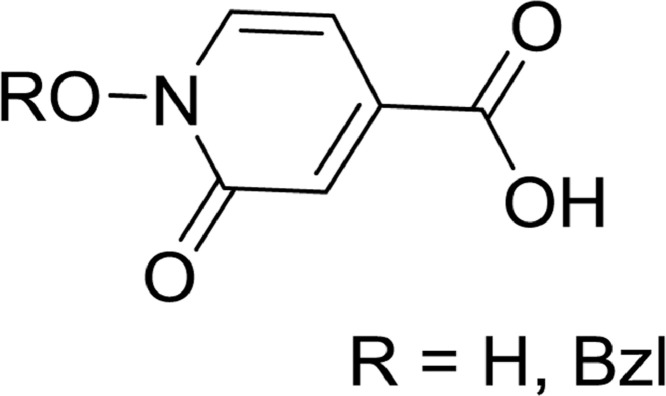

## Specifications table

**Table:** 

Subject Area	Chemistry
More specific subject area	Peptide Synthesis
Method name	-
Name and reference of original method	D. Al Shaer, F. Albericio, BG. de la Torre. Solid-Phase Synthesis of Peptides Containing 1-Hydroxypyridine-2-one (1,2-HOPO). Tetrahedron Lett., (2020), https://doi.org/10.1016/j.tetlet.2020.152299
Resource availability	-

## Method details

**Scheme 1:**
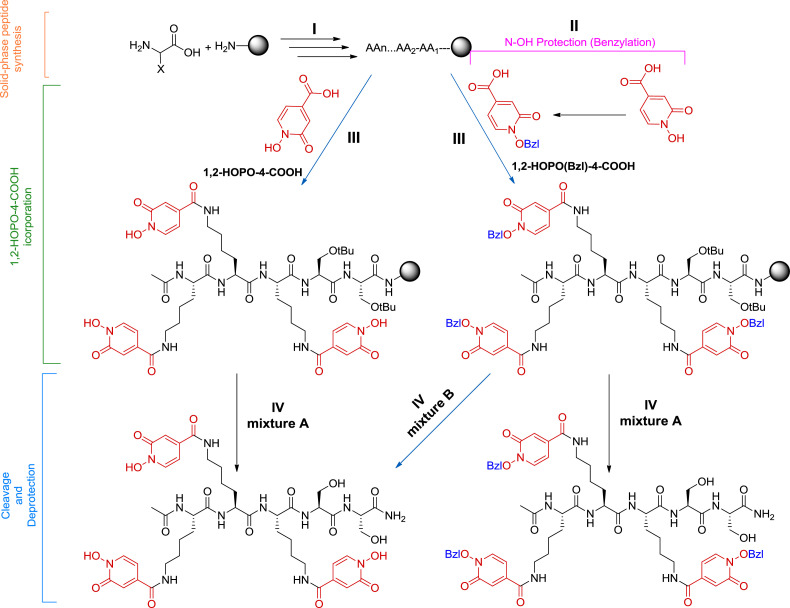
Synthesis of 1,2-HOPO-containing peptides using both strategies. Blue arrows represent the steps that were introduced and/or optimized.
Vacuum pump
*Procedure*

1,2-HOPO containing peptides were synthesized on solid-phase by derivatization of Lys side chain residues once the peptide chain was fully elongated. This derivatization was done in two different routes, the first route uses the free form (no *N-*OH protection) of 1,2-HOPO-4-COOH (peptides 1 and 2, Fig. 2). The second route uses the Bzl protected N-OH form of 1,2-HOPO-4-COOH as it is commonly reported in the literature (peptide 3, Fig. 2). Peptide 1 was obtained in a very good purity, while peptide 2 was obtained in lower but acceptable purity and, very importantly, very easy to be purified. This is due because the main impurities containing only two or one incorporation of 1,2-HOPO-4-COOH moieties, appeared in the HPLC far away of the target peak. In peptide 3, the removal of the Bzl groups is carried out during the global deprotection and cleavage of the peptide from the resin using a friendlier condition [TFA- TFMSA-H_2_O (8:3:1)] than those reported in the literature. Scheme 1 describes all the synthetic steps of both routes for the synthesis of the peptides.

**Fig. 1:**
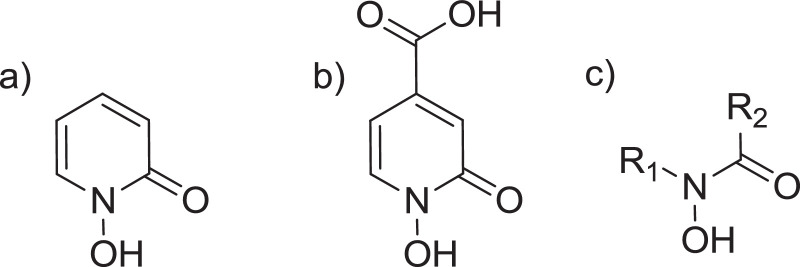
(a) 1,2-HOPO (b) 1,2-HOPO-4-COOH (c) General structure of a hydroxamic acid.

**Fig. 2:**
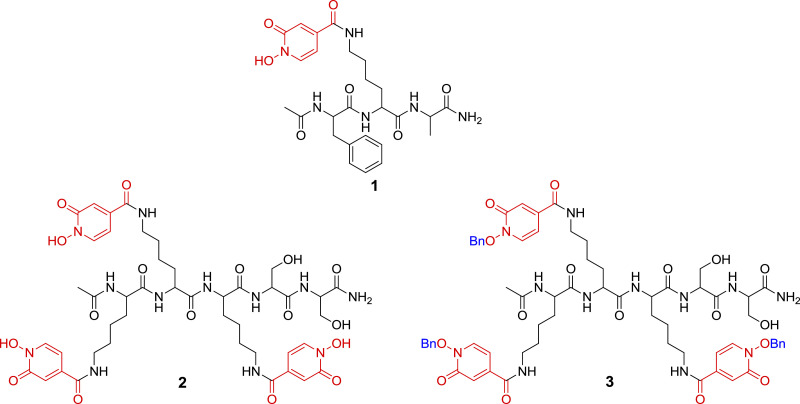
Peptides synthesized containing 1,2-HOPO moieties.

**Fig. 3:**
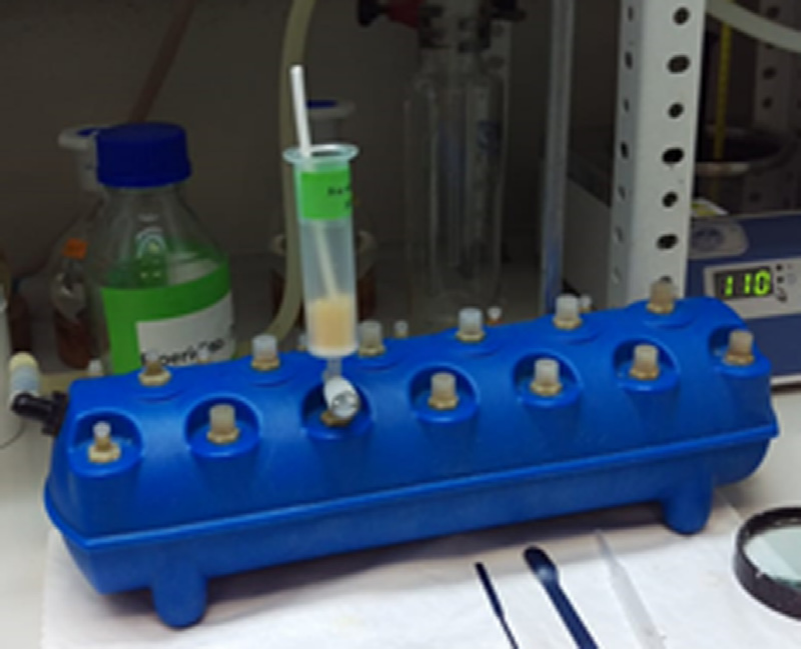
Set up for solid phase peptide synthesis (manifold + syringe + Teflon rod + Teflon stopcock).

The different steps are:

(I) **Peptide backbone elongation on solid support**

(This step was performed following a standard Fmoc/tBu strategy protocol. 0.1 mmol of the peptide will be prepared following these steps:
*Chemicals and apparatus*
***Caution:*** *You should perform steps* ***I-IV*** *in a fume hood wearing a protective coat, goggles, and gloves.*
Fmoc-Rink-amide-AM-PS-resin (Loading: 0.7 mmol/g, Iris Biotech, 200-400 mesh)
N^α^-(9-Fluorenylmethoxycarbonyl) amino acids: Fmoc-Ala-OH, Fmoc-Lys(Mtt)-OH, Fmoc-Phe-OH (for peptide 1), and Fmoc-Ser(tBu)-OH, Fmoc-Lys(Mtt)-OH for peptide 2 and 3. Mtt: methyltritryl, tBu: tert-butyl, Iris Biotech.
N,N’-Diisopropylcarbodiimide (DIC) (Luxembourg Bio Technologies. **Be careful**!!, it is flammable and irritant)
OxymaPure (Luxembourg Bio Technologies)
Acetyl chloride (AcCl) Sigma-Aldrich, **Be careful**!!, it is flammable and corrosive)
N,N-Diisopropylethylamine (DIEA) (reagent grade 99%, Sigma-Aldrich, **Be careful**!!, it is flammable and corrosive)
Piperidine (reagent grade 99%, Sigma-Aldrich, **Be careful**!!, it is flammable, corrosive and toxic)
N,N-Dimethyl formamide (DMF) (extra pure AR grade, SRL, **Be careful**!!, it is flammable, harmful and health hazard)
Analytical balance (Mettler Toledo)
5 mL polypropylene syringe fitted with a polypropylene preinserted frit (20 µm).
Vacuum manifold (Vac-Man® Laboratory Vacuum Manifold, Promega) (Fig. 3)

1. Prepare the syringe fitted with a polypropylene filter.
2. Weigh Fmoc-rink-amide-AM-PS-resin (143 mg for 0.1 mmol synthesis scale) and place it in the syringe.
3. Place the syringe on the manifold (as in Fig. 3) and adjust the suction (you only need a gentle suction).
4. Wash the resin with DMF (1 min, 1 mL) and drain the solvent over gentle vacuum, repeat it twice.
5. Add DMF (3 mL) and allow it to swell the resin for about 15 min. *This step will make the active sites of the resin to be accessible for the reactants in the following steps*.
6. Meanwhile, in separate glass vials, weigh 0.3 mmol (3 eq. with respect to the resin). of each the Fmoc-AA-OH required to build the peptide chain. [To prepare peptide 1, Fmoc-Ala-OH: 93.4 mg, Fmoc-Lys(Mtt)-OH:187.4 mg, and Fmoc-Phe-OH:116.2 mg,. To prepare peptides 2 and 3, 2x Fmoc-Ser(tBu)-OH:115 mg, and of 3x Fmoc-Lys(Mtt)-OH187.4 mg, for each peptide].
7. Add (43 mg, 0.3 mmol) of OxymaPure to each Fmoc-AA-OH vial. *This additive will form the corresponding active ester after that the Fmoc-amino acid has been activated with DIC see step #12, modulating the reactivity to minimize early hydrolysis and/or side-reaction formation. See ref [**7**] for a better understanding of the mechanism of the reaction*.
8. To remove the Fmoc protecting group, prepare a 20% solution of piperidine in DMF. *It is not necessary to prepare freshly this solution. Once per week is advisable, keep it a dark glass bottle.*
9. Once the resin is well swollen, filter off the solvent and start the synthesis.
10. Add the piperidine solution (1 mL) to the resin, stir for 1 min and drain the solution off. Add again 1 mL of the piperidine solution and stir for 7 min, then filter and wash with DMF (3 × 1 ml).
11. Dissolve the mixture of Fmoc-AA-OH/OxymaPure corresponding to the residue to be incorporate (be aware that the peptide synthesis starts by the C-terminal end of the sequence). It is advisable to use the minimum amount of DMF for dissolving the mixture but should be enough to cover the swollen resin (for 143 mg of this kind of resin, approx. 600 µL are needed).
12. To the previous mixture solution, add the coupling reagent, DIC (47 µL, 0.3 mmol) and allow the activation for 2 min. *The active ester is formed. More time could conduct to the hydrolysis of the ester and/or epimerization.*
13. Transfer the solution to the drained resin in the syringe (after Step 10), make sure all resin beads are covered with the solution, add few more drops of DMF if needed.
14. Leave the slurry to react for 40-60 minutes with occasional stirring. The course of the reaction could be confirmed by the ninhydrin test.
15 Drain the solution, wash with DMF (1 mL, 30 sec.) with stirring, drain it, and repeat the washing for two more times.
16. Repeat Steps 8–15 for each amino acid in the sequence.
17. Proceed to Step 10 to remove the last Fmoc protecting group.
18. To acetylate the N-terminal of the prepared peptide, add DIEA (345 µL, 20 mmol), then AcCl (95 µL, 10 mmol), shake it for 30 min., drain the solution and wash with DMF (3 × 1 mL).
(II) **Protecting the N-OH of 1,2-HOPO-4-COOH [1,2-HOPO(Bzl)-4-COOH]**

This step was done following with slight modifications the procedure reported in literature for other 1,2-HOPO derivative [8].
*Chemicals and apparatus*
4-Carboxy-1-hydroxypyridin-2-one (1,2-HOPO-4-COOH) (ChemSpace)
Benzyl bromide (BzlBr) (Sigma-Aldrich)
Potassium carbonate (K_2_CO_3_) (Sigma-Aldrich)
Methanol (MeOH) (Laboratory reagent >99.6%, Sigma-Aldrich)
6 M HCl
Ice
Round bottom flask
Condenser
Oil bath
Hotplate and stirrer
Rotatory evaporator
*Procedure*

1. In a round bottom flask with a magnetic stirrer, weigh 1,2-HOPO-4-COOH (155 mg, 1 mmol) and K_2_CO_3_ (268 mg, 2 mmol).
2. Add 10 mL of MeOH and place the round bottom flask in an oil bath.
3. Stir the suspension and add BzlBr (143 µL, 1.2 mmol) drop wise over 1 min.
4. Attach the condenser, and reflux the mixture at 65–70 °C for 16 h.
5. Remove the MeOH under reduced pressure in a rotatory evaporator.
6. Dissolve the residue with H_2_O (5 mL). If does not readily dissolve add few drops of 1M NaOH.
7. Acidify the aqueous solution with HCl (6 M) dropwise to pH 2, a white precipitate should heavily form.
8. Filter the precipitate, wash it with ice-cold water, and dry it under vacuum.
9. Characterize the product using NMR, analytical HPLC and LCMS. The yield is about 70%.
(III) **Incorporation of HOPO (protected and unprotected) to the peptide**

*Chemicals and apparatus*
Peptidyl resins prepared in part I (Ac-F-K(Mtt)-A-NH-Rink-amide-resin and Ac-K(Mtt)-K(Mtt)-K(Mtt)-S(tBu)-S(tBu)-NH- Rink-amide-resin)
1,2-HOPO-4-COOH (ChemSpace).
1,2-HOPO(Bzl)-4-COOH (prepared in II)
N,N-Diisopropylcarbodiimide (DIC) (Luxembourg Bio Technologies. **Be careful**!!, it is flammable and irritant)
N,N-Dimethyl formamide (DMF)) (extra pure AR grade, SRL, **Be careful**!!, it is flammable, harmful and health hazard)
Trifluroacetic acid (TFA) (reagent grade 98%, Sigma-Aldrich. **Be careful**!!, it is corrosive and harmful)
Triisopropyl silane (TIS) (Reagent grade98%, Sigma-Aldrich. **Be careful**!!, it is flammable).
Dichloromethane (DCM) (ACS reagent >99.9%, Honeywell. **Be careful**!!, it is harmful and health hazard).
Diisopropylethylamine (DIEA) (reagent grade 99%, Sigma-Aldrich. **Be careful**!!, it is flammable and corrosive)
5 mL polypropylene syringe fitted with a polyethylene filter
The solid phase peptide set up mentioned in section I (Fig. 3).
*Procedure*

1. Place the peptide resin in the syringe, place it on the manifold and wash it with DCM (3 × 1 mL)
2. To remove Mtt protecting, prepare a solution of TFA-TIS-DCM (3:5:92) (300 µL TFA, 500 µL TIS, 9.2 mL DCM).
3. Add 2 ml of the TFA solution to the peptide resin and shake it for 15 min. *Evolution and disappearance of yellow color is detected, which indicates the release of the Mtt carbocations and its posterior scavenging*.
4. Filter off the solution. Wash the resin with DCM (3 × 3 mL) for 30 sec.
5. Repeat Steps 3 and 4 for one more time.
6. Wash the resin with 5% DIEA in DCM solution (3 × 1 mL). *The DIEA treatments is to liberate the free amine of the Lys.*
7. Wash the resin with DCM (3 × 1 ml), then with DMF (2 × 1 mL), the peptide resin is now ready for the incorporation of 1,2-HOPO-4-COOH, unprotected and protected.
8. a) Unprotected: dissolve 24 mg (0.15 mmol) of 1,2-HOPO-4-COOH per each Lys to be derivatized, in 600 µL of DMF;

b) Protected: dissolve 37 mg (0.15 mmol) of Bzl protected 1,2-HOPO-4-COOH per each Lys to be derivatized, in 600 µL of DMF.

1. Transfer the HOPO solution to the resin, then add DIC (24 µL, 0.15 mmol per Lys), shake for 90 min, drain the solution off, then wash with DMF (3 × 1 mL).
2. An optional second coupling can be done using less equivalents of HOPO and DIC to increase the yield (as in Table 1). But as 1,2-HOPO-4-COOH is rather expensive, this step can be avoided.
3. In case of using the unprotected HOPO (in peptides 1 and 2), treat the peptidyl resin with 1 mL of 20% piperidine in DMF solution for 10 min. (*To remove any ester oligomers that may have formed through the N-OH).* Wash the resin with DMF (3 × 1 mL)
4. Wash the resin with DCM (3 × 1 mL).
(IV) **Cleaving the peptide from the resin**

*Chemicals and apparatus*
Peptide resins prepared in part III (Ac-F-K(HOPO)-A-NH-resin, Ac-K(HOPO)-K(HOPO)-K(HOPO)-S(tBu)-S(tBu)-NH-Rink-amide-resin and Ac-K(HOPO-Bzl)-K(HOPO-Bzl)-K(HOPO-Bzl)-S(tBu)-S(tBu)-NH-Rink-amide-resin)
Trifluoromethanesullfonic (TFMSA) (reagent grade 98%, Sigma-Aldrich. Be careful!!, it is corrosive and harmful)
Trifluroacetic acid (TFA) (reagent grade 98%, Sigma-Aldrich. **Be careful**!!, it is corrosive and harmful)
Triisopropyl silane (TIS) (Reagent grade98%, Sigma-Aldrich. **Be careful**!!, it is flammable).
Dichloromethane (DCM) (ACS reagent >99.9%, Honeywell. **Be careful**!!, it is harmful and health hazard).
Diethylether (DEE)
Methanol
Milli-Q water
5 mL polypropylene syringe fitted with a polyethylene filter
Falcon tube
Two cleavage mixtures were used:
**Mixture A** (TFA-TIS-H_2_O, 95:2.5:2.5) to cleave peptide from resin and to remove the acid labile protecting groups, namely, tBu from the Ser side chain (and leaving the Bzl unaffected if it is present) (this mixture was used for all peptides).
**Mixture B** (TFA-TFMSA-TIS, 8:3:1) to cleave the peptide from the resin and to remove all other protecting groups including the Bzl (this mixture was used to get peptide 2 from **peptide resin** 3).
*Procedure*

1. Place the peptide resin in the syringe and wash it with MeOH and dry under gentle suction.
2. Weigh the resin and transfer it into a falcon tube fitted with a cap.
3. Add the cleavage mixture (1 mL/100 mg peptidyl resin), close the cap and shake the tube for 70 min.

For **mixture A**:

1. Add ice-cold diethyl ether (5 mL/1 mL cleavage mixture), shake well (the peptide will precipitate), centrifuge the mixture and decant the supernatant, repeat the ether washing for two more times. *Do not discard the diethyl ether washing till you get your peptide at the end.*
2. Dry the residue under reduced pressure, add water (3 mL) to dissolve the peptide.
3. Filter off the resin beads and keep the filtrate.
4. Wash the resin beads two more times with (1 mL) water each, filter and combine all filtrates.
5. Lyophilize the peptide solution.

For **mixture B**

1. Add water (5 mL/1 mL cleavage mixture).
2. Wash the aqueous layer with diethyl ether (5 mL).
3. Remove the ether layer and repeat the washing 2 more times.
4. Filter off the resin beads.
5. Lyophilize the aqueous filtrate.
(V) **Purification and Characterization**

All peptides were purified using reversed phase semi preparative HPLC, the pure fractions were lyophilized and stored at 4 °C. All peptides were obtained in good yields. They were characterized using analytical HPLC and LCMS techniques. Purities of peptides were determined by comparison of peak area for each peptide in the HPLC chromatogram at 220 nm. Yields were determined from the mass of the lyophilized peptide in relation with the theorical mass (see Table 1).

**Table 1:** Purities and yields for the prepared peptides, 1-3.

Peptide	Form of HOPO incorporated	Cleavage solution used	Purity	% Yield
1	Free N-OH	Mixture A	92% for single coupling (1.5 eq) 97% for double coupling (1.5 eq, then 0.75 eq)	88% crude peptide
2	Free N-OH	Mixture A	39% for single coupling (1.5 eq per Lys) 60% for double coupling (1.5 eq per Lys, then 0.75 eq per Lys)	87 % crude peptide
3	N-OBn	Mixture A	69%	71 % crude peptide
2 from 3	N-OBn	Mixture B	62%	63 % crude peptide

*Note:* HPLC and MS-LC chromatograms can be found in the original report [9].
